# Environmental metagenetics unveil novel plant‐pollinator interactions

**DOI:** 10.1002/ece3.10645

**Published:** 2023-11-07

**Authors:** Sydney B. Wizenberg, Laura R. Newburn, Rodney T. Richardson, Mateus Pepinelli, Ida M. Conflitti, Mashaba Moubony, Daniel Borges, M. Marta Guarna, Ernesto Guzman‐Novoa, Leonard J. Foster, Amro Zayed

**Affiliations:** ^1^ Department of Biology York University Toronto Ontario Canada; ^2^ Appalachian Laboratory University of Maryland Center for Environmental Science Frostburg Maryland USA; ^3^ Ontario Beekeepers' Association Tech‐Transfer Program, Orchard Park Office Centre Guelph Ontario Canada; ^4^ Beaverlodge Research Farm, Agriculture and Agri‐Food Canada Beaverlodge Alberta Canada; ^5^ School of Environmental Sciences University of Guelph Guelph Ontario Canada; ^6^ Department of Biochemistry & Molecular Biology and Michael Smith Laboratories Vancouver British Columbia Canada

**Keywords:** bees, metabarcoding, metagenomics, pollen, pollination

## Abstract

Honey bees are efficient pollinators of flowering plants, aiding in the plant reproductive cycle and acting as vehicles for evolutionary processes. Their role as agents of selection and drivers of gene flow is instrumental to the structure of plant populations, but historically, our understanding of their influence has been limited to predominantly insect‐dispersed flowering species. Recent metagenetic work has provided evidence that honey bees also forage on pollen from anemophilous species, suggesting that their role as vectors for transmission of plant genetic material is not confined to groups designated as entomophilous, and leading us to ask: could honey bees act as dispersal agents for non‐flowering plant taxa? Using an extensive pollen metabarcoding dataset from Canada, we discovered that honey bees may serve as dispersal agents for an array of sporophytes (*Anchistea*, *Claytosmunda*, *Dryopteris*, *Osmunda*, *Osmundastrum*, *Equisetum*) and bryophytes (*Funaria*, *Orthotrichum*, *Sphagnum*, *Ulota*). Our findings also suggest that honey bees may occasionally act as vectors for the dispersal of aquatic phototrophs, specifically *Coccomyxa* and *Protosiphon*, species of green algae. Our work has shed light on the broad resource‐access patterns that guide plant‐pollinator interactions and suggests that bees could act as vectors of gene flow, and potentially even agents of selection, across Plantae.

## Graphical abstract

We used pollen metabarcoding to investigate if honey bees interact with and disperse cells from non‐flowering plants. We discovered that honey bees may serve as dispersal agents for an array of sporophytes (*Anchistea*, *Claytosmunda*, *Dryopteris*, *Osmunda*, *Osmundastrum*, *Equisetum*) and bryophytes (*Funaria*, *Orthotrichum*, *Sphagnum*, *Ulota*). Our findings also suggest that honey bees may occasionally act as vectors for dispersal of aquatic phototrophs, specifically *Coccomyxa* and *Protosiphon*, species of green algae. These findings, though preliminary, open up a new field of research.

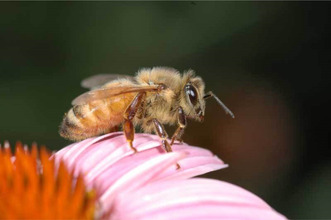

## 1 INTRODUCTION

**FIGURE 1:**
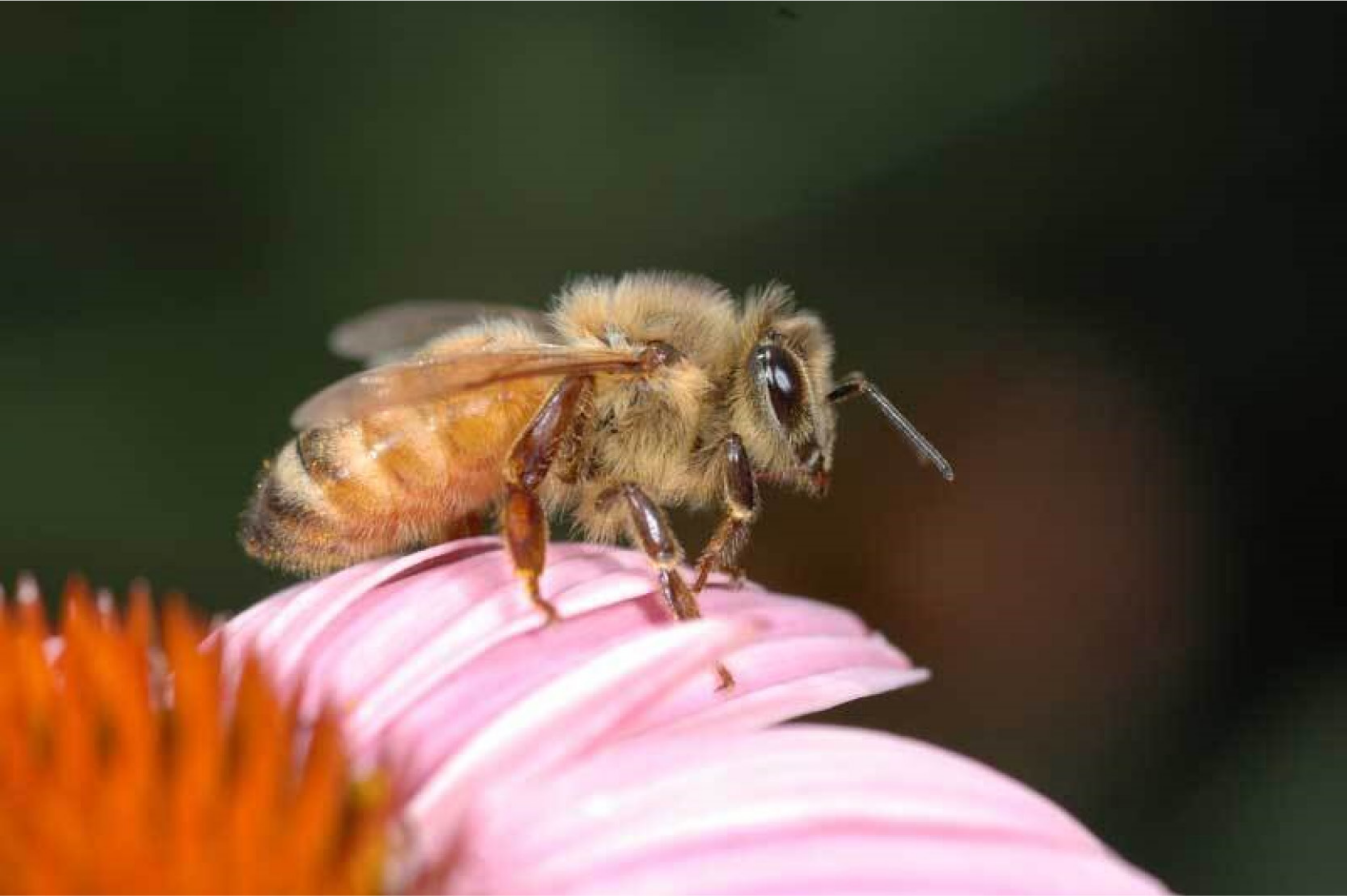
Honey bee worker visiting a purple coneflower (*Echinacea purpurea*). Photograph by A. Zayed.

Pollinators are influential to the structure of flowering plant populations – they act as agents of selection, and drivers of gene flow, impacting the evolutionary trajectory of many sessile species (Cresswell, 2003; Gervasi & Schiestl, 2017; Ramos & Schiestl, 2019). Their role as vectors for the dispersal and transmission of pollen grains is fundamental to the structure of terrestrial ecosystems, but our understanding of their importance has historically been limited to flowering plants that rely on entomophily. Within these taxa, pollinators may influence the direction and pace of selection, acting as vehicles for evolutionary processes and determinants of the vegetative composition of their habitat. Though many invertebrates act as vectors for the dispersal of pollen (Ghisbain et al., 2021; Macgregor et al., 2015; Prather et al., 2013), honey bees (*Apis mellifera*) are among the most prolific and highly generalized (Hung et al., 2018; Klein et al., 2007; Requier et al., 2019), making them ideal study systems for exploring plant‐pollinator interactions (Figure 1). Their capacity to impact plant population structure has been a topic of interest for many years, and recent advancements in the study of plant‐pollinator ecology have shed light on the complex dynamics surrounding this mutualism (Elliott et al., 2021; Richardson et al., 2021; Sponsler et al., 2020). Pollen metabarcoding has presented a new frontier for rapid high‐throughput analysis of plant‐pollinator interactions, utilizing conserved barcoding regions of the plant genome to accurately identify the abundance and diversity of plant species in mixed pollen samples (Bell et al., 2016, 2022). With this advancement has come a plethora of new data that suggest that dietary breadth of honey bees may be wider than originally believed (Lau et al., 2019; MacIvor et al., 2014; Richardson et al., 2015; Stoner et al., 2022), and thus, honey bees may exhibit a stronger influence over their environment than previously thought.

Due to their sessile nature, plants rely on external vectors for dispersal of gametophytes; they are incapable of physically finding a mate, so they depend on abiotic or biotic forces to facilitate the fusion of gametes. Their intergenerational life cycle results in ephemeral haploid gametophytes that act as links between parental plants and their offspring – dispersal of these specialized cells is essential for reproductive success (Robledo‐Arnuncio et al., 2014; Snow & Lewis, 1993; Wizenberg et al., 2022). Most plant species rely on either anemophily (wind pollination) or entomophily (insect pollination) as a dispersal mechanism, frequently evolving specialized pollen morphology to maximize success under one of those two conditions (Lu et al., 2022; Pacini & Franchi, 2020; Wizenberg et al., 2020). Though the predominant form of pollen dispersal often guides the evolution of sex characteristics, categorization of dispersal strategies is not rigid, and many plant species may simultaneously engage in both biotic and abiotic dispersal. This has most often been seen in anemophilous species; detection of plant taxa that typically rely on wind dispersal is common in pollen metabarcoding datasets (Chiara et al., 2021; De Vere et al., 2017; Pornon et al., 2016). Metagenetic detection of plant families like Oleaceae and Fagaceae (Richardson et al., 2015, 2019), known to be predominantly anemophilous (Kaul, 1986; Wallander, 2008), has suggested that honey bees may possess greater dietary breadth than originally believed. This has highlighted the power they hold as generalists and raises the question: could honey bees be acting as dispersal agents across Plantae? Using a substantial pollen metabarcoding dataset, we set out to explore if pollen samples collected by honey bees in Southern Ontario contained cells associated with previously undocumented non‐flowering plant taxa.

## 2 MATERIALS AND METHODS

### 2.1 Pollen collection

**FIGURE 2:**
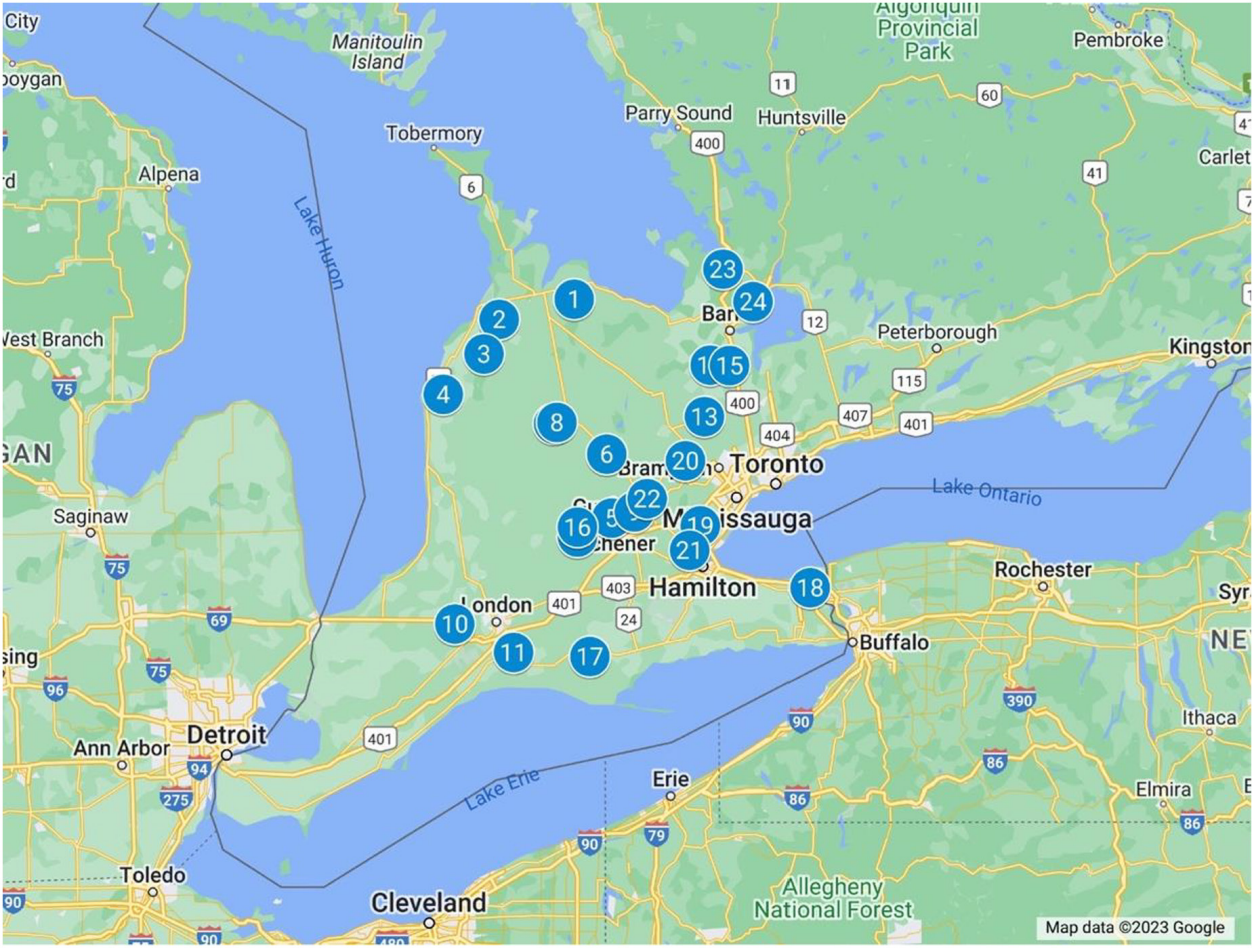
Study sites in Ontario, Canada. Each site had a minimum of 1 colony and a maximum of 3. Pollen was collected in the form of bee bread from each site on a seasonal basis (spring, summer, fall). Image created with GoogleMaps.com.

We collected this data as part of a larger project focused on understanding honey bee health and seasonal pollen foraging patterns in Southern Ontario (Canada). In partnership with local beekeepers and the Ontario Beekeepers Association, we placed 50 honey bee colonies at 24 sites in Southern Ontario (Figure 2) and sampled pollen from each colony three times to capture seasonal foraging patterns. The three time points represented spring (late May – early June), summer (July), and fall (late August – early September). We do not provide exact GPS coordinates for apiaries to protect the identity of the beekeepers and farmers involved; site descriptions are available upon reasonable request. For each sampling event, we collected approximately 15 g of “bee bread” from each hive – pollen that had been previously collected, brought back to the hive, and undergone processing by worker bees. Bee bread is stored as small pellets, compressed together as a heterogenous mixture of pollen, nectar, bee saliva, bacteria, and yeast (Khalifa et al., 2020). We harvested the bee bread by selecting hive cells that contained recently collected pollen, distinguishable by the light degree of compaction, using a 7″ birch wood stir stick to remove the clumped pollen and transfer it into a sterile 50 mL conical tube. A half‐full 50 mL collection tube averaged approximately 15 g of pollen. After collection, we sealed each pollen sample and brought it back to the lab at York University, where we stored the samples at −80°C until undergoing analysis.

### 2.2 Pollen metabarcoding

We utilized our previously validated pollen metabarcoding protocol (Wizenberg et al., 2023). We extracted DNA from 10 g of bee bread using the NucleoMag DNA Food Kit (Macherey‐Nagel); we combined each pollen sample with 20 mL of lysis buffer: 70% autoclaved filtered water (Millipore Sigma), 20% 10× STE (100 mM NaCl, 10 mM Tris, 25 mM EDTA), and 10% diluted SDS (10% sodium dodecyl sulfate). We inverted each sealed sample 10 times and then created a homogenized suspension by shaking each sample for 10 min in an orbital shaker (G25 Incubator Shaker, New Brunswick Scientific) at 25°C and 375 rpm. We then pipetted 3 mL of the sample into a 7 mL bead beater tube (Bead Mill 24, Fisherbrand) containing 10 small (1.4 mm) and 2 large (2.8 mm) ceramic beads, bead‐beat it for 2 min total (4 × 30 s), and transferred 550 μL of the homogenized suspension to a 1.5 mL Eppendorf tube. We warmed CF lysis buffer for 10 min in a 65°C water bath then added 550 μL of the warmed buffer and 10 μL of Proteinase K to the Eppendorf tube containing the homogenized sample and vortexed (Mini Vortex Mixer, VWR) for 30 s. We then incubated the sample at 65°C for 30 min in a block heater (Isotemp 145D, 250 V, Fisherbrand), inverting every 10 min. After incubation, we added 20 μL of RNase A (New England Biolabs) and allowed the sample to incubate at room temperature (20°C) for an additional 30 min. After incubation, we centrifuged the sample for 20 min at 21,900 RCF (Centrifuge 5810 R, 15 amps, Eppendorf), transferred 400 μL of the upper liquid layer to the binding plate, added 25 μL of NucleoMag B‐Beads and 600 μL of binding buffer CB (both from the NucleoMag DNA Food kit) then ran an extraction program (KingFisher Flex, Thermo Scientific). Each of the 5 deep well plates used to complete the extraction program contained either 600 μL of CMW buffer (wash 1), 600 μL of CQW buffer (wash 2.1), 600 μL of 80% EtOH (wash 2.2), or 100 μL of buffer CE (elution). After the extraction program was complete, we transferred 80 μL of the eluted sample to a fresh 1.5 mL Eppendorf tube.

We performed two PCR programs, the first of which amplified the region of interest (PCR1), and the second of which extended the target sequence with a read priming sequence (PCR2), for each of the two primers (ITS2, rbcL1) employed as part of our metabarcoding program (Table 1). For each PCR program, we used 96 well plates, containing 84 pollen samples, six negative controls, and six positive controls (Banana, *Musa* sp.). We pipetted 11 μL of sterile H_2_O, 12.5 μL of 2× Taq Pol Mix (New England Biolabs), 0.5 μL of each relevant forward and reverse primer (1 μM), and 0.5 μL of sample DNA (~36.2 ng) into each well. We then ran a PCR cycle (Eppendorf Mastercycler, Ep Gradient) using the following program specifications: initial denaturation (94°C, 10 min, 1 cycle), denaturation (94°C, 30 s, 40 cycles), primer annealing (54°C, 40 s, 40 cycles), extension (72°C, 1 min, 40 cycles), final extension (72°C, 10 min, 1 cycle). PCR1 product was used as the template for PCR2; the same master mix described above was combined with 0.5 μL of PCR1 product. Program specifications were maintained between PCR1 and PCR2, with one change to the primer annealing temperature (56°C). Following PCR2, we prepared samples for Illumina Sequencing by performing a third PCR program that tagged each sample with a unique combination of forward and reverse indexes; PCR3 program specifications follow that described above, with a primer annealing temperature of 60°C. We then normalized the resulting PCR3 product using a SequalPrep Normalization Kit (Invitrogen) and shipped the normalized samples on dry ice for Illumina Sequencing at Genome Quebec.

**TABLE 1:** PCR1 and PCR2 primer specifications.

Primer	Sequence
PCR1	
rbcL1 (F)	5′‐AGACCTWTTTGAAGAAGGTTCWGT‐3′
rbcL1 (R)	3′‐TCGCATGTACCTGCAGTAGC‐5′
ITS2 (F)	5′‐ATGCGATACTTGGTGTGAAT‐3′
ITS2 (R)	3′‐TCCTCCGCTTATTGATATGC‐5′
PCR2	
rbcL1 (F)	5′‐**CAGCGTCAGATGTGTATAAGAGACAG**AGACCTWTTTGAAGAAGGTTCWGT‐3′
rbcL1 (R)	3′‐**GCTCGGAGATGTGTATAAGAGACAG**TCGCATGTACCTGCAGTAGC‐5′
ITS2 (F)	5′‐**CAGCGTCAGATGTGTATAAGAGACAG**ATGCGATACTTGGTGTGAAT‐3′
ITS2 (R)	3′‐**GCTCGGAGATGTGTATAAGAGACAG**TCCTCCGCTTATTGATATGC‐5′

*Note*: (F) indicates a forward primer, and (R) indicates the paired reverse primer. Bolded values are changes to the forward and reverse primers between PCR1 and PCR2.

### 2.3 Bioinformatics

All data processing was completed in Python (v. 3.10.7), and R (v. 4.2.1), using the *dada2* (Callahan et al., 2016; v. 1.16.0, 2020‐04‐07) and *purrr* (Henry & Wickham, 2020; v. 0.3.4, 2020‐04‐16) packages. We processed returned sequence data by first pairing forward and reverse reads, trimming primer sequences, and grouping identical sequences under unique ASVs (amplicon sequence variants). We then built a database that linked species to ASVs associated with each genetic marker using the MetaCurator method (Richardson et al., 2020). We used this database to parse through returned sequence data and identify the species associated with each ASV, setting a precursory condition of >0.9 similarity. We used negative controls as indicators of mistagging frequency and filtered real sample data to remove detections with a high likelihood of representing mistag‐associated false detections (Richardson, 2022). After reviewing the resulting metagenetic data, we filtered out observations associated with seed‐bearing taxa (angiosperms and gymnosperms), leaving 37 ASVs. For each resulting ASV, we used NCBI's BLAST (basic local alignment search tool), to confirm that the taxa associated with each ASV was the closest sequence hit across all reference databases. To rule out potential contamination of samples, we also sequenced both our negative and positive controls. Notably, all 37 ASVs associated with non‐flowering taxa were only detected in real samples, and not in any of the positive or negative controls that underwent sequencing, ruling out the risk of lab contamination as a source of false discovery. All resulting ASVs that underly the findings presented below are provided as Appendix S1.

## 3 RESULTS

After filtering out all sequence hits associated with angiosperm and gymnosperm species and blasting all remaining sequences through the NCBI database to confirm sequence identification, we found 37 ASVs (amplicon sequence variants) associated with plant taxa of the orders Coccomyxaceae (*incertae sedis*), Protosiphonales, Osmundales, Polypodiales, Equisetales, Sphagnales, Orthotrichales, and Funariales. This indicated the presence of three novel dietary constituents – phytoplankton, sporophytes, and bryophytes (Table 2). All 37 ASVs associated with non‐flowering plant taxa were detected using a single biomarker (*rbcL*).

**TABLE 2:** Non‐flowering plant taxa detected in pollen samples from Southern Ontario.

Genus	% ASV‐ref	# seq‐hits	# pollen samples	Seasonality
Phytoplankton				
*Coccomyxa*	91.2 (±0.6)	8	2	Spring, fall
*Protosiphon*	95.6	2	1	Fall
Sporophytes				
*Anchistea*	99.5	2	1	Summer
*Claytosmunda*	99.7	66	2	Summer
*Dryopteris*	99.7	9	1	Summer
*Osmunda*	96.0 (±4.0)	19	4	Spring, summer
*Osmundastrum*	99.5 (±0.5)	163	11	Spring, summer
*Equisetum*	99.3 (±1.3)	145	13	Spring, summer, fall
Bryophytes				
*Funaria*	99.7	2	1	Spring
*Orthotrichum*	99.5	2	1	Fall
*Sphagnum*	99.5	4	1	Fall
*Ulota*	96.9	2	1	Spring

*Note*: % ASV‐ref is the proportional similarity between amplicon sequence variants (ASVs) in the metagenetic dataset and the equivalent reference sequence on NCBI, # Seq‐hits is the frequency of occurrence of that ASV across all 150 pollen samples, # pollen samples is the number of samples associated with that summed sequence hit.

Four genera of bryophytes were identified via our bioinformatics pipeline, with an average sequence match of 98.9% (±1.3); *Orthotrichum*, *Ulota*, *Sphagnum*, and *Funaria*. Sporophyte species associated with two distinct groups, ferns and horsetails, were abundant in our filtered dataset. All 13 ASVs linked to horsetail species were from the same genera, *Equisetum*, with an average sequence match of 99.3% (±1.3). ASVs associated with fern species were much more common, encompassing five different genera across 17 ASVs. The most abundant fern genus, *Osmundastrum*, had an average sequence match of 99.5% (±0.5). The second most abundant fern genus, *Osmunda*, had an average sequence match of 96% (±4.0); the remaining fern genera (*Anchistea*, *Claytosmunda*, and *Dryopteris*) were all associated with singular ASVs and an average sequence match of 99.6% (±0.1). The three remaining ASVs were linked to *Coccomyxa* and *Protosiphon*, genera of green algae, with an average sequence match of 92.7% (±2.6).

**FIGURE 3:**
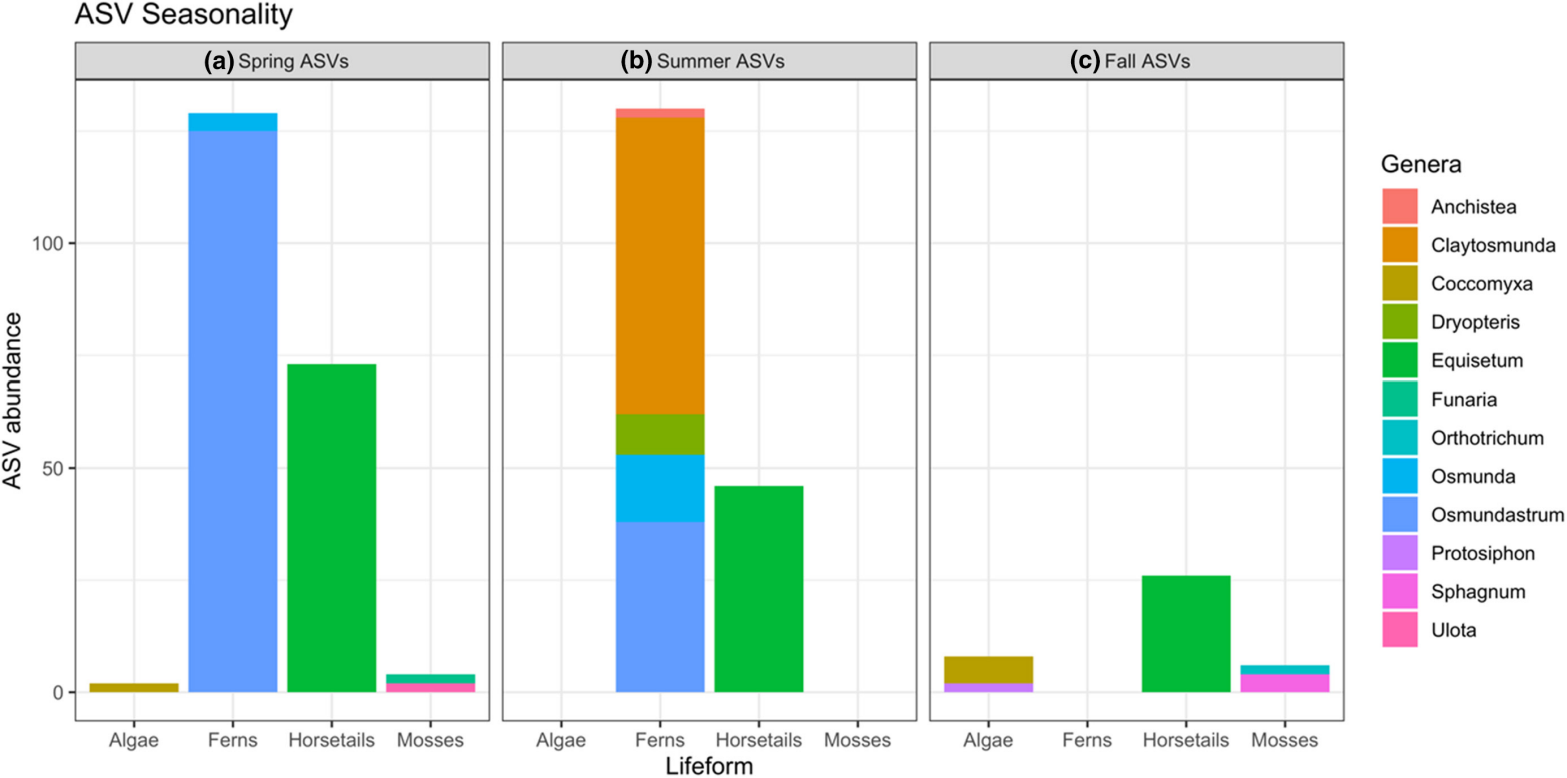
Seasonality of ASV (amplicon sequence variant) distribution. Seq‐hit abundance indicates the number of reads associated with that lifeform detected during each season. Colors indicate the genera diversity of each lifeform.

Time point 1 samples, which were collected during the spring, were associated with the detection of genera from all three novel lifeforms (phytoplankton, sporophytes, and bryophytes), including multiple species of ferns and mosses (Table 2, Figure 3). Time point two samples, which were collected during the summer, were abundant in cells associated with fern genera (*Osmundastrum*, *Dryopteris*, *Osmunda*, *Anchistea*, *Claytosmunda*) and horsetails (*Equisetum*), but not any species of moss nor algae (Table 2, Figure 3). Time point 3 samples, which were collected during the fall, included cells associated with all three novel lifeforms (*Coccomyxa*, *Protosiphon*, *Equisetum*, *Orthotrichum*, *Sphagnum*), but with lower sporophyte diversity than spring samples (Table 2, Figure 3). Spring pollen samples contained the greatest number of novel plant taxa sequence hits (208), from the greatest number of pollen samples (13). Summer pollen samples contained a similar number of novel plant taxa sequence hits (176) from a lower number of pollen samples (10). Fall pollen samples contained the lowest number of novel plant taxa sequence hits (40) across the lowest number of samples (6). Across all three seasons, non‐flowering plant taxa were detected in pollen samples from 23 colonies at 14 unique sites. For most colonies, non‐flowering plant taxa were only detected in pollen samples from a single time point. Notably, ASVs associated with non‐flowering plant taxa occurred in very low abundance overall, representing a variable but small (<1%) proportion of our honey bees' diets.

## 4 DISCUSSION

**FIGURE 4:**
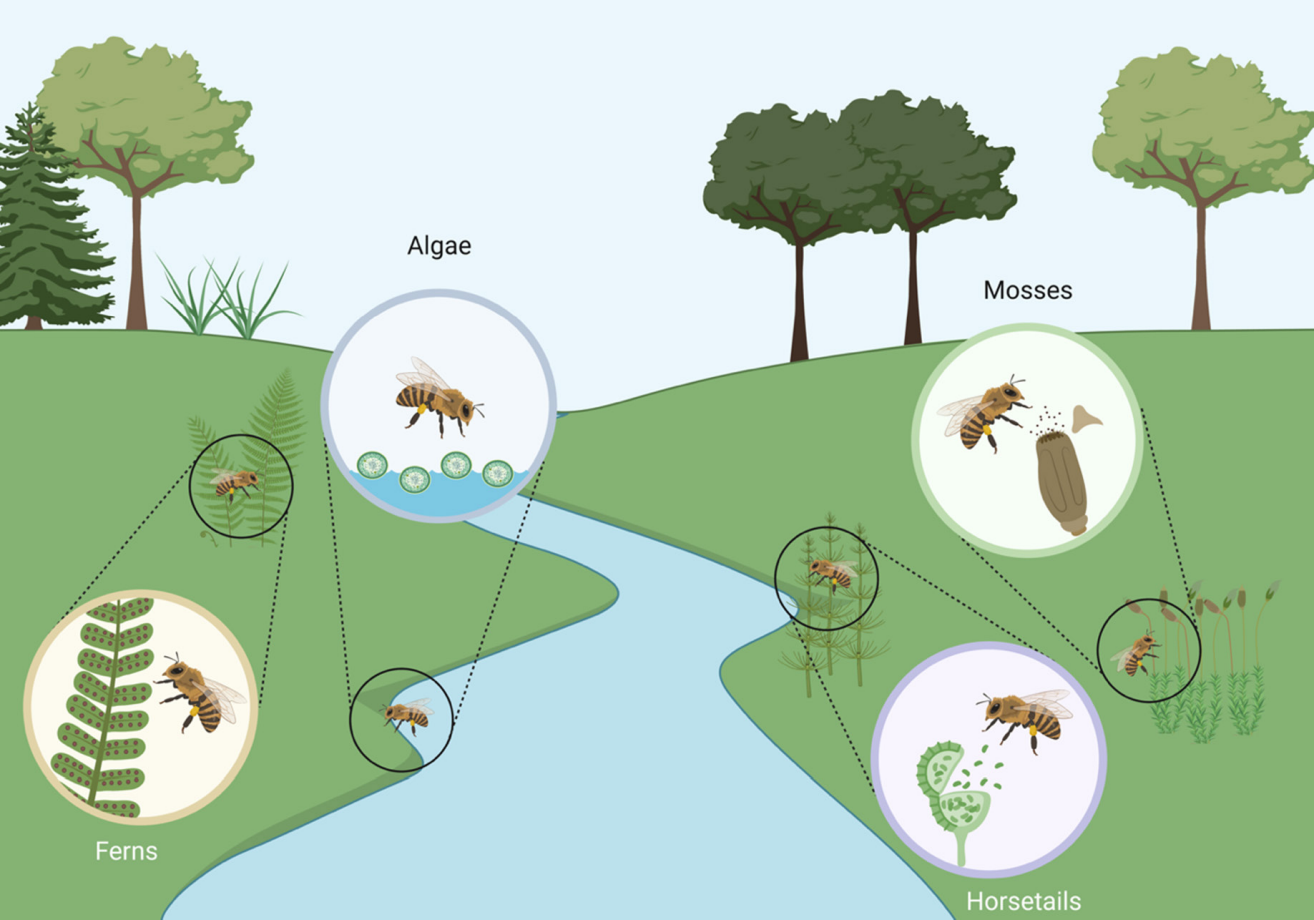
Honey bees interact with a diverse array of non‐flowering plants. Conceptual image created with BioRender.com.

The presence of cells associated with bryophytes, sporophytes, and phytoplankton species in our pollen samples suggests that we may have detected previously undocumented resource access patterns (Figure 4). Though they occurred in low abundance overall, the high sequence matches for species of ferns, horsetails, and mosses provide a degree of confidence in our discovery. To our knowledge, no previous pollen metabarcoding projects have publicly documented the presence of a diverse array of algae, moss, or horsetail cells in their pollen samples. Fern spores have been found in honey samples dating back as far as 3300 BC (Kvavadze et al., 2007), and as recently as 2015 (Carmen Seijo et al., 2011; Hawkins et al., 2015; Moar, 1985; Olga et al., 2012), implying that interactions between sporophytes and honey bees may span back millennia. Earlier work relying on observational data found that honey bees interacted with ferns, despite no detectable nectar production (Olesen, 1988) – suggesting that these resource‐access patterns may be intentional, as opposed to coincidental. More recent work that relied on a pollen metabarcoding approach for exploring plant‐pollinator interactions found that ferns are underrepresented in ITS metabarcoding reference databases (Banchi et al., 2020), which may explain why they were only detected using our second marker (*rbcL*). Gous et al. (2021) suggest that fern spores in particular may occur consistently in pollen metabarcoding datasets, but always in low abundance, thus they may have been previously overlooked (Gous et al., 2019).

Documentation of these interactions between species of moss and insect pollinators is novel, but some work suggests that it may be beneficial. Compounds found in bryophytes have sporicidal properties and might reduce the severity of larval diseases experienced by honey bees, but these antibacterial benefits may be limited to the refined format that has undergone pharmacological processing (Karaoğlu et al., 2023). Very little work has been done on understanding the potential benefits of phytoplankton as a dietary constituent; Ricigliano et al. (2020) found that cyanobacteria was a good source of nutrition for honey bees, and it may provide access to nutrients not typically found in terrestrial food webs (Lenihan‐Geels et al., 2013). Algae is an abundant source of omega‐3 fatty acids, alpha‐linolenic acid (ALA), eicosapentaenoic acid (EPA), and docosahexaenoic acid (DHA), key compounds for the physiological function of vertebrates (Hixson et al., 2015). Omega‐3 deficiencies have been shown to impact learning in invertebrates (Arien et al., 2015), and honey bee colonies deficient in these key nutrients have been shown to alter their patterns of communication to emphasize foraging success when identifying floral resources abundant in missing nutrients (Zarchin et al., 2017). Empirical work in Lepidoptera has demonstrated that over‐saturation of omega‐3 fatty acids can adversely alter development (Hixson et al., 2016), suggesting that diets abundant in algae may not be invariably optimal. This may explain the overall low abundance of algae cells in our pollen samples; if algae is providing access to key nutrients, but over‐saturation induces adverse developmental effects, foraging may be regulated by individual requirements and represent a low dietary constituent when a colony is in good health.

Though we cannot determine if interactions with non‐flowering plants are intentional or coincidental, in this context, the motivation behind pollinator‐mediated transmission of non‐flowering plant taxa is inconsequential. Long‐distance dispersal events, regardless of the rarity or stochasticity, can strongly influence the evolutionary ecology and genetic diversity of newly established populations (Darwin, 1859; Nathan, 2006; Ridley, 1930; Robledo‐Arnuncio et al., 2014; Wu et al., 2022). While it remains unclear if honey bees exhibit any foraging preferences in relation to non‐flowering species, they hold power in their ability to influence biogeographical processes (Aavik & Helm, 2018; Gillespie et al., 2012; Wu et al., 2022). Anecdotally, we have seen honey bees from our colonies interacting with bryophytes as a source of water (S2), suggesting that these interactions are intentional. Though more work is needed, our research suggests that honey bees, and likely other bees, could play an important role as vectors for gene flow, if not also as agents of selection, for non‐flowering plant species. Although these discoveries are interesting, they are limited in their geographic scope. Replication of these findings across a broader range of regions and years may provide more insight into the complex web of interactions that underpin the structure of terrestrial ecosystems, and the role of pollinators as vehicles for evolutionary processes across Plantae.

## AUTHOR CONTRIBUTIONS

**Sydney B. Wizenberg:** Conceptualization (lead); data curation (lead); formal analysis (lead); investigation (lead); methodology (equal); writing – original draft (lead); writing – review and editing (equal). **Laura R. Newburn:** Methodology (equal); writing – review and editing (equal). **Rodney T. Richardson:** Methodology (equal); resources (equal); software (equal); writing – review and editing (equal). **Mateus Pepinelli:** Investigation (equal); writing – review and editing (equal). **Ida M. Conflitti:** Funding acquisition (equal); project administration (lead); resources (equal); writing – review and editing (equal). **Mashaba Moubony:** Investigation (equal); writing – review and editing (equal). **Daniel Borges:** Investigation (equal); writing – review and editing (equal). **M. Marta Guarna:** Funding acquisition (equal); investigation (equal); resources (equal); writing – review and editing (equal). **Ernesto Guzman‐Novoa:** Funding acquisition (equal); investigation (equal); resources (equal); writing – review and editing (equal). **Leonard J. Foster:** Funding acquisition (equal); investigation (equal); resources (equal); writing – review and editing (equal). **Amro Zayed:** Conceptualization (lead); funding acquisition (lead); investigation (lead); resources (lead); supervision (lead); writing – review and editing (equal).

## CONFLICT OF INTEREST STATEMENT

The authors declare no competing interests.

## Supporting information


Appendix S1:

Video S1:

## Data Availability

All metagenomic data underlying the findings are available as Appendix S1.
