# Supplementary material for: Environmental metagenetics unveil novel plant‐pollinator interactions
Source: Ecol Evol. 2023 Nov 7;13(11):e10645. doi: 10.1002/ece3.10645 (PMC10630067; doi:10.1002/ece3.10645)
Supplement: Supplementary file 2 — Video S1: [file ECE3-13-e10645-s001.zip › S2_video-caption.docx]

**S2 Caption**: A honey bee from a rooftop urban colony in Toronto, Ontario, interacting with moss as a source of water. Video captured by SB Wizenberg.
